# Urethral Reconstruction Using Mesothelial Cell-Seeded Autogenous Granulation Tissue Tube: An Experimental Study in Male Rabbits

**DOI:** 10.1155/2017/1850256

**Published:** 2017-02-28

**Authors:** Shiwei Jiang, Zhonghua Xu, Yuanyuan Zhao, Lei Yan, Zunlin Zhou, Gangli Gu

**Affiliations:** ^1^Department of Urology, Qilu Hospital, Shandong University, Jinan, China; ^2^Department of Propaganda, Shandong Provincial Traditional Chinese Medical Hospital, Jinan, China

## Abstract

*Objective*. This study was to evaluate the utility of the compound graft for tubularized urethroplasty by seeding mesothelial cells onto autogenous granulation tissue.* Methods*. Silastic tubes were implanted subcutaneously in 18 male rabbits, of which nine underwent omentum biopsies simultaneously for in vitro expansion of mesothelial cells. The granulation tissue covering the tubes was harvested 2 weeks after operation. Mesothelial cells were seeded onto and cocultured with the tissue for 7 days. A pendulous urethral segment of 1.5 cm was totally excised. Urethroplasty was performed with mesothelial cell-seeded tissue tubes in an end-to-end fashion in nine rabbits and with unseeded grafts in others as controls. Serial urethrograms were performed at 1, 2, and 6 months postoperatively. Meanwhile, the neourethra was harvested and analyzed grossly and histologically.* Results*. Urethrograms showed cell-seeded grafts maintained wide at each time point, while strictures formation was found in unseeded grafts. Histologically, layers of urothelium surrounded by increasingly organized smooth muscles were observed in seeded grafts. In contrast, myofibroblasts accumulation and extensive scarring occurred in unseeded grafts.* Conclusions*. Mesothelial cell-seeded granulation tissue tube can be successfully used for tubularized urethroplasty in male rabbits.

## 1. Introduction

Urethral reconstruction remains a great challenge for urologists. Complete resection of unhealthy urethral tissues is necessary for the procedure. When the urethral lesion is extensive, a larger urethral gap will be created and need to be bridged with tubular grafts. Currently, the urethral reconstruction is usually undertaken by tubularizing the urethral plate, foreskin, bladder, or buccal mucosa [1–4]. However, short- or long-term complications occur commonly, including anastomotic strictures, diverticulum, fistula, stone formation, hair growth, and donor site morbidities [5–9]. Besides, suturing patch graft into tubular graft around urethral catheter is time-consuming. It has also been concerned that the presence of longitudinal suture line might potentially increase formation of urethrocutaneous fistula [10]. To simplify the procedure, natural tubular grafts were investigated for urethral reconstruction, such as autologous vein and urethral acellular matrix [11, 12]. However, harvesting vein grafts with suitable diameter is difficult and traumatic. In addition, urethral replacement using urethral acellular matrix yields poor results.

It has been reported that a granulation tissue tube of any required diameter can be produced by planting silicone mandrel subcutaneously [13], which provides potentially an autogenous natural tubular graft for urethral reconstruction. Several studies have shown that an epithelial coverage of tubular graft is necessary for healthy regeneration of the urethra without subsequent stricture due to its function as permeability barriers between the graft and urine [14–16]. Urothelial cells (UCs) [14, 15], epidermal cells [16], and oral keratinocytes [17, 18] are commonly used to construct epithelial lining for the graft. However, low proliferative abilities of epithelial cells are concerned. It often requires a long incubation period before the cells populate the full matrix, and the formed engineered urethra can be used as a graft for implantation. Besides, cells are not available with malignant conditions, a history of lichen sclerosis, or oral disease.

Accumulating results have been reported to successfully use mesothelium-lined grafts as urethral grafts, including peritoneum [19] and tunica vaginalis [20]. Previously, we have demonstrated that mesothelial cells (MCs) can play roles of seed cells for urethral reconstruction [21]; therefore, MCs might be proper substitutes for epithelial cells as speculated. MCs show higher proliferation ability than UCs do. Besides, harvesting MCs from omentum is much easier with less chance of cell pollution than harvesting UCs from urinary tract. In this study, we seeded MCs onto the autogenous granulation tissue to construct a mesothelium-lining compound graft to investigate whether it could be successfully used for long-segmental tubularized urethroplasty in male rabbits.

## 2. Materials and Methods

### 2.1. Subcutaneous Tubing Implantation and Harvest

All animal experiments were approved by Animal Care and Use Committee of Shandong University (Shandong, China). Eighteen male New Zealand white rabbits with weight 2.5–4 kg (mean 3.2 kg) aged from 6 to 8 months were used. A small ventral midline incision was made on the skin. Subcutaneous tissue bilateral to the incision was dissected by using surgical scissors. Based on the diameter of male rabbit urethra, we chose 10 Fr silastic tube (Chensheng, Shandong, China) as a mold. A total of four 3 cm long tubes were implanted for each animal. Tubes were placed inside the subcutaneous tissue and sutured to dermis at one end with 6/0 polypropylene (Prolene). The skin incision was closed with 3/0 polyglactin 910 (Vicryl). Penicillin G sodium (10^5^ U/day) was administered intramuscularly for 3 days after operation.

**Figure 1:**
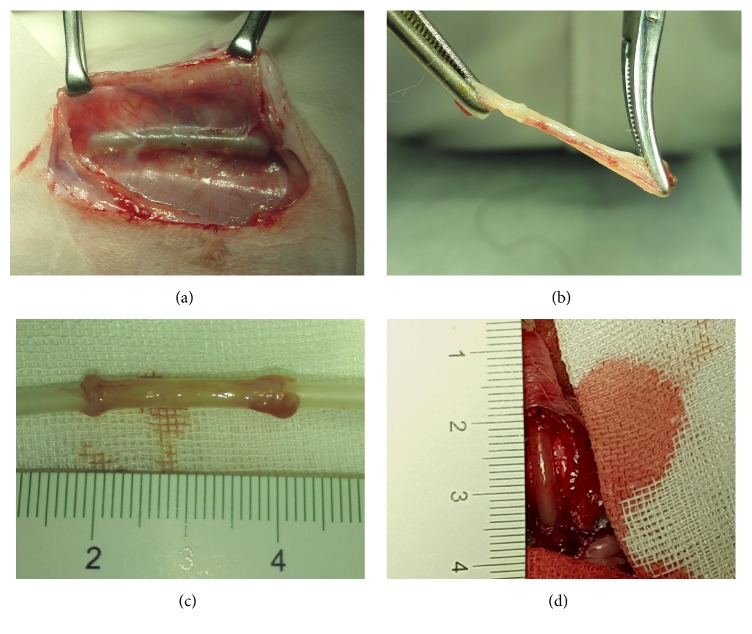
(a) Silastic tube was completely encapsulated by granulation tissue 2 weeks after implantation. (b) Harvested tissue with good tensile strength was demonstrated. (c) Tissue was trimmed to 2 cm length before transplantation. (d) Tubularized urethroplasty with the tissue was performed.

When the silastic tubes become encapsulated by autologous granulation tissue 2 weeks after implantation (Figures 1(a)–1(c)), the animals were anesthetized to reopen the subcutaneous tissue via original incision. One implant wrapped with the thickest tissue was harvested for construction of compound graft, or as a control of direct urethral reconstruction. Other implants were used for histological and electron microscopical analysis.

### 2.2. Harvesting, Culture, and Identification of MCs

Omentum biopsies were simultaneously procured from 9 of the 18 rabbits with subcutaneous tubing implantation. Peritoneal cavity was opened to aseptically harvest a 2 × 2 cm biopsy specimen of omentum. The omentum specimen was washed with phosphate buffered saline (PBS) 3 times and trimmed to remove fat and blood vessels as much as possible. Under continuous agitation in 0.125% trypsin/0.01% EDTA (Gibco, Grand Island, NY) at 37°C for 15 mins, MCs were dissociated from the specimens. The isolated MCs were then cultured onto 25 mL culture plates with low glucose Dulbecco's modified Eagle's medium (DMEM) plus 20% fetal bovine serum (FBS) (Gibco, Grand Island, NY). The cells were expanded to a density of 2 × 10^6^ cells/cm^2^ before seeding. Phenotypic analysis of cultured cells was determined by immunofluorescent staining using antibodies of pancytokeratin AE1/AE3 (Santa Cruz Biotechnology, Dallas, TX) and vimentin (Abcam, Cambridge, UK), respectively.

### 2.3. Construction of the Compound Tubular Graft

Upon the third passage, MCs were trypsinized with 0.25% trypsin/0.02% EDTA and collected to be centrifuged at 1500 rpm for 5 mins. The cell pellets were resuspended in fresh DMEM. The silastic tube was slipped out of the tubular tissue to discard. The harvested tubular tissue was washed 3 times in PBS and trimmed to 2 cm long. A new aseptic segment of 10 Fr silastic tube was reinserted to support the tubular tissue. MCs were then homogeneously seeded onto the outer surface of the tissue with DMEM solution at a density of 2 × 10^6^ cells/cm^2^. The cells were allowed to settle and adhere to the tissue for 4 h, followed by addition of enough medium. Before being used for urethral replacement, the compound graft was incubated at air-liquid level in DMEM for 7 days. The culture medium was refreshed every 2 days. Random samples of the seeded grafts were obtained for histological and transmission electron microscopical analysis. The unseeded tubular tissue was incubated in DMEM for 24 h as a control.

### 2.4. Transmission Electron Microscopy of the Compound Grafts

The seeded grafts were trimmed to 1 mm × 1 mm × 3 mm, fixed immediately with 3% glutaraldehyde, and postfixed with 1% osmium tetraoxide. The specimen was stained en bloc with 0.5% uranyl acetate and embedded in Epon 812. Thin sections were stained and examined under a JEOL-1200EX microscope (JEOL, Japan).

### 2.5. Urethral Surgery and Postoperative Evaluation

Nine of the 18 rabbits received MCs-seeded compound grafts, and the other 9 rabbits received unseeded grafts as controls. Before implantation, the compound grafts were gently everted to let the mesothelium layer turn to the lumen and store in DMEM temporarily. A 1.5 cm segment of penile urethra was separated circumferentially and excised. To reduce tensile stress, the grafts were trimmed to 2 cm, interposed, and anastomosed with interrupted sutures by using 6/0 Vicryl on both ends. Two nonabsorbable sutures using 6/0 Prolene were left on both ends to mark the edges of the graft (Figure 1(d)). An 8 Fr urethral catheter was maintained for 14 days postoperatively. Cervical collars were used to prevent animals from removing the urethral catheter. Penicillin G sodium (10^5^ U/day) was administered intramuscularly for 5 days postoperatively.

Every 6 animals were euthanized at the first, second, and sixth postoperative month, respectively. Retrograde urethrograms were performed to assess the urethral caliber, and then the entire urethra was gently removed. The urethral lumen was cut longitudinally, and the marking sutures were identified for gross examination and histological analysis.

### 2.6. Histological Analysis of the Grafts and Retrieved Urethra

Specimens were fixed with 10% buffered formalin and then paraffin-embedded. Transverse sections of the seeded and unseeded grafts were prepared for hematoxylin-eosin staining (H&E) and immunohistochemistry analysis. Antibodies of pancytokeratin AE1/AE3 and vimentin were used to detect MCs. Alpha-smooth muscle actin (*α*-SMA) antibody (Abcam, Cambridge, UK) was used to detect myofibroblast cells.

To dynamically evaluate the neourethra regeneration process, longitudinal sections of the junctional and central part of the retrieved urethra were prepared for H&E staining, Masson's trichrome, and immunohistochemistry analysis, respectively. Pancytokeratin AE1/AE3 antibody was used to detect urothelium. *α*-SMA antibody was used to detect smooth muscle fibers. Immunolabeling was performed by using avidin-biotin detection system. The sections were counterstained with hematoxylin.

## 3. Results

**Figure 2:**
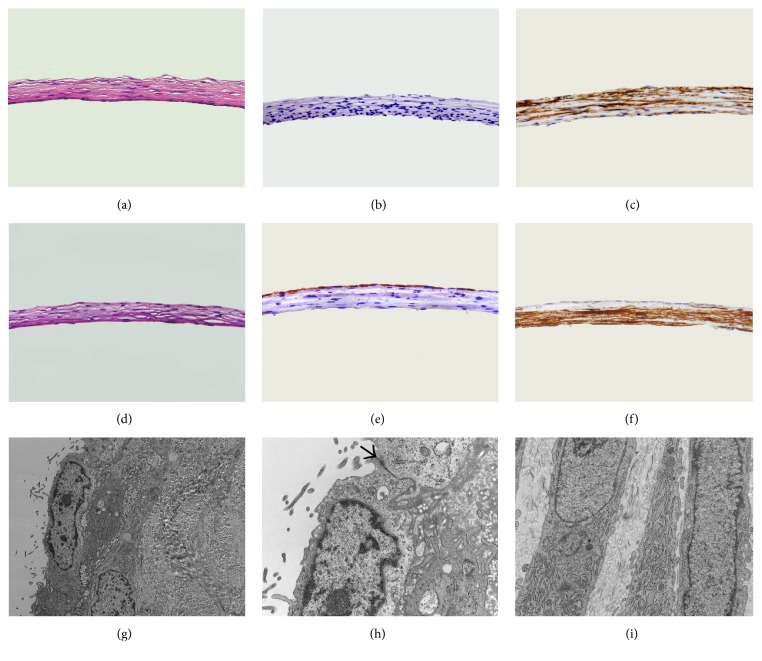
Histological analysis of the tissue are shown by (a) H&E staining of the unseeded graft (400x), (b) immunostaining for cytokeratin AE1/AE3 of the unseeded graft (400x), (c) immunostaining for *α*-SMA of the unseeded graft (400x), (d) H&E staining of the seeded graft (400x), (e) immunostaining for cytokeratin AE1/AE3 of the seeded graft (400x), (f) immunostaining for *α*-SMA of the seeded graft (400x), (g) transmission electron micrographs of MCs on the surface of graft (10000x), (h) tight junction between neighboring MCs indicated by an arrow (30000x), and (i) transmission electron micrographs of myofibroblasts (15000x).

All animals survived after surgery. Two weeks after implantation, all tubes were encapsulated with autologous granulation tissues (Figure 1). Histological analysis of the tissue demonstrated the composition of myofibroblasts embedded in concentric layering of collagen bundles (Figure 2). As shown in transmission electron micrograph, the spindle-shaped myofibroblasts contained large amounts of synthetic organelles (Figure 2(i)).

**Figure 3:**
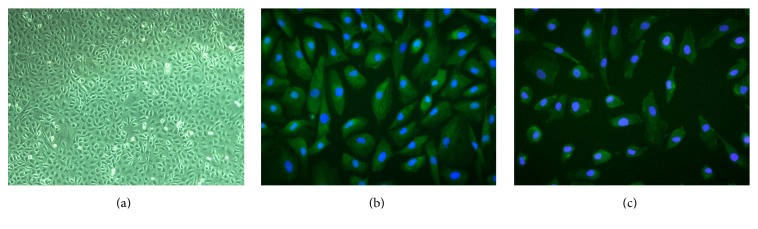
Identification of the in vitro culture of MCs is shown by (a) phase contrast photomicrograph of cultured cells at second passage (100x), (b) immunofluorescent staining for cytokeratin AE1/AE3 of MCs (400x), and (c) immunofluorescent staining for vimentin of MCs (400x).

The image of phase contrast microscope showed that MCs maintained cobblestone-like growth pattern at the third passage, which was similar to epithelial cells (Figure 3(a)). MCs with positive expression of AE1/AE3 and vimentin were also demonstrated by using immunofluorescent staining (Figures 3(b) and 3(c)). Histological analysis of the MCs-seeded compound graft showed a single layer of mesothelium formed on the outer surface of the tissue tube after incubation for 7 days (Figure 2(e)). The characteristic surface microvilli and tight junctions of MCs were proved by transmission electron microscopy (Figure 2(h)).

**Figure 4:**
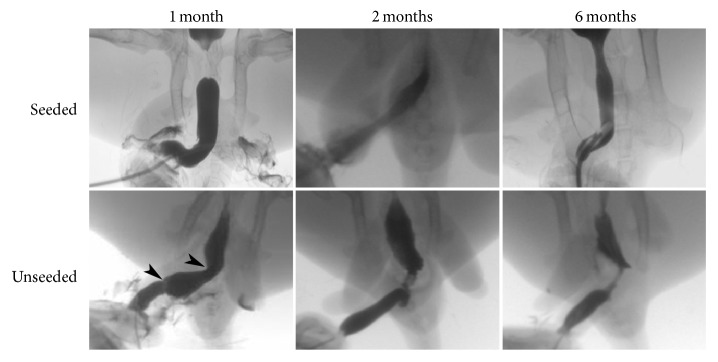
Retrograde urethrograms of both seeded and unseeded grafts at 1, 2, and 6 months after operation. Arrowheads indicate mild stricture formed at the end-to-end anastomosis in the unseeded groups.

Urethral catheters were removed 2 weeks after urethral reconstruction. Serial urethrograms were performed for assessment of urethral caliber. In the unseeded group, mild stricture formation was observed at the end-to-end anastomosis at 1-month after implantation, and remarkable strictures of entire replaced segment occurred at 2 and 6 months after implantation (Figure 4, bottom). In contrast, the neourethra remained patent at each time period of the seeded group (Figure 4, upper).

**Figure 5:**
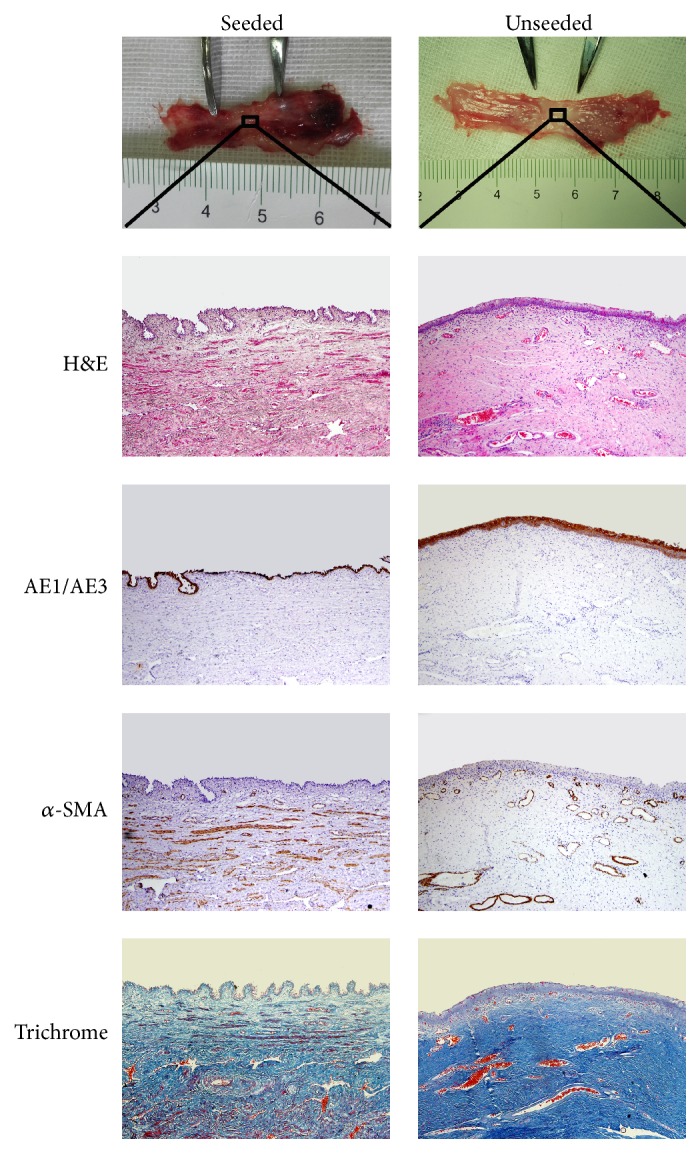
Gross and histological examinations of the retrieved urethra 6 months after implantation. In the seeded grafts, the mucosa appeared normal with no severe shrinkage as indicated by the marking sutures (upper). In the unseeded grafts, severe fibrosis occurred. Histological analysis showed layers of urothelium and organized smooth muscle bundles in the seeded grafts and extensive scarring of collagen fibers in the unseeded grafts (original magnification 100x).

**Figure 6:**
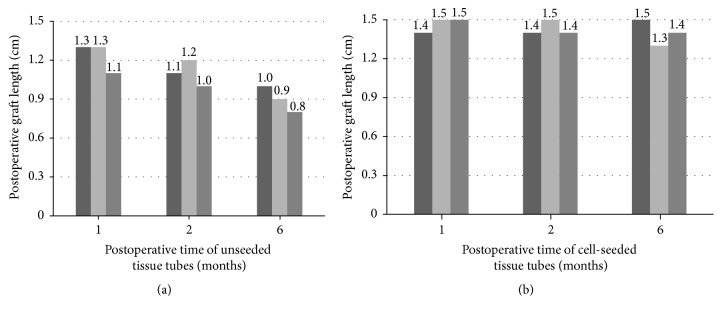
Postoperative graft length at each time point. (a) The graft length was decreased over time in the unseeded group. (b) No severe shrinkage was observed in the seeded group.

Gross examination of the unseeded grafts revealed severe fibrosis and contracture of the urethral lumen with pale and stiff mucosa (Figure 5). Progressive graft shrinkage was observed in the unseeded group (Figure 6(a)). However, the mucosa of the neourethra appeared normal without fibrosis or ulcer formation (Figure 5), and no severe shrinkage was observed as indicated by marking sutures in the seeded grafts (Figure 6(b)).

**Figure 7:**
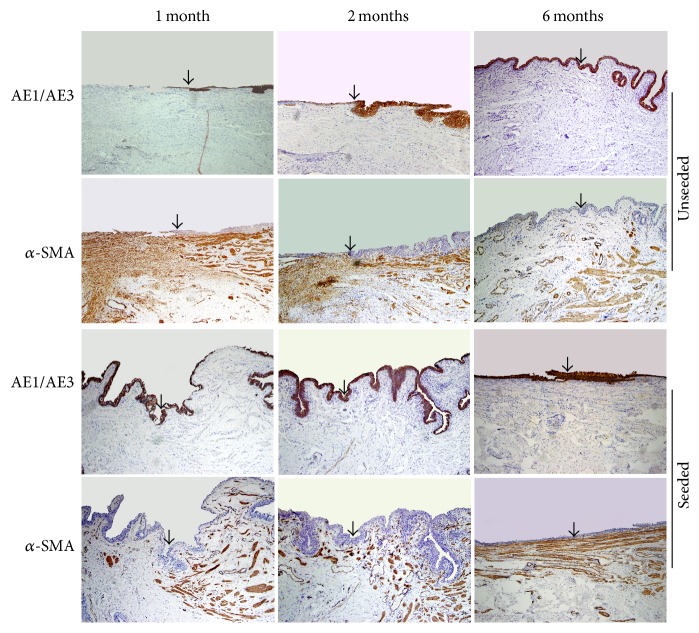
Histological analysis of the margin between native urethra (upper layer) and the graft. In the unseeded grafts, discontinuous urothelial layer and myofibroblasts accumulation within the graft were observed at 1 month postoperatively. Intact urothelial layer formed and myofibroblasts accumulation were still observed at 2 months. Myofibroblasts disappeared and extensive scarring formed at 6 months. In the seeded grafts, intact urothelial layer formed at 1 month postoperatively. Smooth muscle cells were observed to infiltrate the grafts. The density of smooth muscle bundles within the grafts increased in a time-dependent manner. The grafts were completely integrated into native urethra by 6 months (original magnification 100x). The arrows indicate the margin between native urethra and the graft.

Histological analysis of the unseeded groups showed discontinued epithelial cells on the surface of the grafts at 1 month after implantation (Figure 7). In addition, large amounts of myofibroblasts were observed with no smooth muscle bundles and capillaries formation within the grafts (Figure 7). At 2 months after implantation, although the grafts were completely covered by multilayers of urothelium, accumulation of myofibroblasts was still observed beneath the urothelium (Figure 7). At 6 months after implantation, the myofibroblasts beneath the well-developed urothelium disappeared and were completely replaced by extensive scarring mainly composed of collagens (Figure 7). Although new capillaries formed, no smooth muscle bundles were observed within the grafts (Figure 5).

Conversely, we observed that the seeded grafts were covered with continuous epithelial layers at 1 month after implantation. The epithelium stained positively with antibody of AE1/AE3 (Figure 7), but negatively with antibody of vimentin (data not shown), implying the newly formed urothelium had completely replaced the original mesothelium. Beneath the urothelium, we observed that neovascularity was well-developed, and disordered smooth muscle bundles migrated from the native urethra into the graft at the end-to-end anastomosis (Figure 7). Sparse muscle bundles were found at the central region of the grafts. No signs of excessive myofibroblasts accumulation, chronic inflammation, and fibrosis were noted. The smooth muscle bundles became increasingly organized and evenly distributed throughout the grafts by 6 months (Figure 5).

## 4. Discussion

A novel method was designed to let the animals grow their own tubular grafts for urethral repair. We utilized a normal biological process whereby foreign objects were placed subcutaneously to induce inflammatory responses and then become encapsulated by myofibroblast-rich granulation tissues. Several advantages were provided in this study. First, they were totally autogenous and immune compatible. Second, they were available for tubularized urethroplasty and could be tailored to various dimensions by altering the size of molding. Third, they exhibited good tensile strength and great resistance to suturing [13]. Finally, the viability of the grafts was maintained by diffusion of nutrients in the subcutaneous tissue due to no vasculature inside, suggesting they might be more adaptable to the initial ischemic microenvironment once implanted in vivo as urethral grafts.

In the present study, we further investigated whether MCs could be used to construct an “epithelial” lining for the granulation tissue tube. The autogenous tissue is a living graft and rich in collagens with bioactive binding sites for cell attachment. Furthermore, its dense architecture makes it suitable to form an “epithelial” coverage. Therefore, we suggest the autogenous tissue possesses essential conditions for cell seeding. Histological inspection confirmed the tissue tube supported the growth of MCs with a layer of mesothelium formed on the outer surface. Tight junctions were also observed between neighboring MCs to serve as impermeable barriers to fluid [22]. We demonstrated that the seeded MCs successfully prevented graft fibrosis and contracture. Besides, much faster regeneration of urothelium in the seeded grafts than that in the unseeded grafts after implantation was observed. However, it is still unclear how the MCs facilitate urothelium regeneration. Increasing evidence supports that MCs have the capacity to differentiate into different phenotypes [23–25]. It is possible that MCs undergo metaplasia to turn into a multilayer urothelium by contact with urine [19]. However, it might also be that MCs just sloughed off the grafts and were gradually replaced by local urothelium. The destiny of MCs after implantation will be further investigated by tracing MCs with 5-bromo-2′-deoxyuridine (BrdU) labeling.

In addition to urothelium, the compound grafts also successfully guided smooth muscle regeneration and gradually remodeled to normal urethral architecture by 6 months. The origin of smooth muscle cells has not yet been determined. Myofibroblasts resemble smooth muscle cells both ultrastructurally and functionally. It has also been demonstrated that cyclical stretch can induce myofibroblasts transformation into smooth muscle phenotype [26, 27]. After implantation, the myofibroblasts underwent repeated stretch during urine emptying. It raises the possibility that smooth muscle cells within the neourethra come from the original myofibroblasts. However, no smooth muscle bundles were observed within the unseeded grafts. Therefore, we suggest the cells were probably derived from native urethral ingrowth. Other groups have also shown that native smooth muscle cells can migrate and regenerate across a distance of 1–1.5 cm [16, 28]. On the other hand, the lower rate of urothelium regeneration and infiltration of urine in the unseeded graft might impede myofibroblasts from transforming into smooth muscle cells. Therefore, the transdifferentiation hypothesis cannot be fully excluded. Furthermore, in urethral tissue engineering, fibroblast is commonly used as a second seed cell to enhance the mechanical properties of the graft, accelerate the regenerative progression, and avoid the recurrence of strictures [29, 30]. We suggest the presence of myofibroblasts in the granulation tissue tube might also have a similar positive effect on the repair.

There are some advantages of our method compared with traditional methods using synthetic or decellularized scaffolds. Two kinds of seed cells, epithelial cells and smooth muscle cells [14], or fibroblasts [18], are needed when using synthetic or decellularized scaffolds to construct a composite urethral graft. The autogenous granulation tissue tube already has a contractile wall of myofibroblasts; therefore, only one kind of seed cells is needed to construct an epithelial lining. This reduced the difficulties of cell seeding, shortened the construction period, and improved the success rate, considering both mesothelial cells and myofibroblasts can grow in DMEM medium. In addition, during preparation of decellularized scaffolds, chemical corrosion and physical oscillation might cause damage to extracellular matrix and loss of growth factors, leading to reduced mechanical property and biocompatibility. And retention of the cellular compounds within the acellular matrix might also cause chronic immunoreactions. Oppositely, as a living tissue with myofibroblasts and collagens, our grafts were strong enough to hold sutures well. The growth factors and cellular binding sites in the collagen fibers were also preserved better to promote cell adhesion and ingrowth. Furthermore, synthetic scaffolds are relatively stiff and unwieldy. It is also concerned that their toxicity, biodegradation rate, and biocompatibility to the host might influence the results of urethral repair and regeneration. Comparatively, the autogenous grafts have better handling characteristics for surgery with good biocompatibility.

Although some novel findings were shown, there were several limitations in this study. First, the urethra defect was created in normal healthy urethra, which could not fully simulate the fibrotic urethra bed in clinical situation. Second, the number of experimental animals is small. Larger sample sizes need to be collected to precisely evaluate the success rate. Finally, rabbit is a small animal model and less informative for clinical problems. Larger animals will also be evaluated in the future. And considering clinical translation, the patients might feel uncomfortable at the implanted area with pains and limited activities. The method also has potential risk of pyogenic infection at the implanted area, especially for patients with diabetes mellitus or immune deficiency, which may lead to failure of the procedure.

## 5. Conclusion

In summary, we successfully constructed a kind of compound tubular grafts with an inner mesothelium, and a contractile wall of myofibroblasts and collagens. They were readily available for tubularized urethroplasty and could guide urethra regeneration with a length of 1.5 cm in male rabbits. Human scrotum might be a suitable option for tube implantation, which resembles subcutaneous tissue of rabbits with fat tissue deficiency. It might become a promising technique in the clinical use for urethral reconstruction in human after further investigations.
